# Cholera

**Published:** 1893-08

**Authors:** Ernest Hart

**Affiliations:** London


ATLANTA

### MEDICAL AND SURGICAL JOURNAL.

Vol. X. AUGUST, 1893. No. 6.

EDITED BY

LUTHER B. GRANDY, M. D., AND MILLER B. HUTCHINS, M. D.,

WITH THE CO-OPERATION OF

H. V. M. MILLER, M. D., LL. D., VIRGIL O. HARDON, M. D.,

FLOYD W. McRAE, M. D., AND ARTHUR G. HOBBS, M. D.

### *ORIGINAL COMMUNICATIONS.*

### CHOLERA.*

By MR. ERNEST HART,

London.

In dealing with the subject of cholera, I am dealing with one which has been, perhaps, more written about, more discussed and more talked about than any other in our time. It is the subject of almost universal conversation. Since I have been here questions have been put to me not only daily but almost hourly. A distinguished gentleman I have seen here to-day reminded me, in our early text-books, the knowledge of cholera was summarized in about these words: "Cholera: Diagnosis, easy; etiology, uncertain; causation, unknown; treatment, useless." Well, now we have modified all that. If I had to take those headings

*Abstract of an address delivered before the American Medical Association, June 8, 1893.

to-day, I think you would agree with me in saying this: Diagnosis, difficult, very difficult. It is only capable of diagnosis by bacillar examination. Now, as to the pathology. The pathology I will not stop with to-day. The pathology has now been immensely cleared up, and the pathology of cholera is now no longer mysterious. We know how to deal with it as we do our other bacteriological diseases. Treatment, useless. We know no more of the treatment of cholera now than we knew of the treatment of cholera thirty years ago. And that is no reproach to us, because for poisons you may find antidotes or partial antidotes, but an infective disease must run its course. We know now there is such a thing as an evolution of disease. Cholera is one of those diseases which the public like to think of as a disease which comes by providence and goes by drugs. That is exactly the opposite of the facts. Cholera comes by man; it is a man-created disease, and cholera can not be cured by drugs. Of course, there are drugs which alleviate and diminish the symptoms. Possibly there are drugs which diminish the number of deaths. But, as at the last Hamburg epidemic, in the early stage and in the later stages of epidemics, when patients are going to get well, everything will cure, and that is the time to try specifics.

But, in the essential periods, when the epidemic is at its height, no drugs will modify the mortality. Now then, as to the causation. We are entitled to say in some parts of Europe cholera will never appear again, and with certain precautions here cholera never can appear again. Now, let me take you briefly over the history and analysis of the great epidemics of cholera since 1853, in order to prove from that precisely the point I am endeavoring to bring to your notice. You know our first outbreak of cholera in Europe was in 1832. We know, first of all, it is epidemic and not endemic. We know it always come from one place. That is India. It is within the imperial British domains of India cholera is always found. In the earlier outbreaks from India, cholera usually came across the great seas, across the Caucasus, and then by caravans and emigrants reached other towns, and finally that ill-fated city, Hamburg. And then, through Hamburg to Europe in general. The other route was by the Mecca pilgrims, the India pilgrims who carried cholera with them to Mecca, and there by a process I will show you diffused it throughout the whole of that country, and then brought it on to Alexandria. From Alexandria it took ship and came to Havre or came to England. That is the course it has pursued now on twenty-five different occasions. And, I notice this: When people travel on foot, on horseback, by caravans, cholera travels correspondingly slow with them. It took three years to come from Odessa to Europe. And, as steamships and railways have increased, its speed has increased. Cholera, then, travels along the lines of human intercourse and precisely with the rapidity of human intercourse. The next point is, that cholera, when we come to examine the facts, is a disease which is always carried by dirty people to dirty places and diffused by dirty water. Now, let us take the places to which it was carried and see what is the demonstration I can offer you as to its being carried by dirty water. Well, we will take, first of all, as an example, London. When cholera first came to London everything was mystery. It was in 1851. We did not know how it came. We thought it was blown by the winds, carried by pandemic waves, and we heard of cholera mists, telluric and meteoric influences and all that jargon. Then came one or two more instances. There was a celebrated pump on Broadway; it was celebrated, and the water most beautiful and sparkling. But, water sparkles from human nitrates. Well, the Broad street pump was a celebrated pump, and people were sending from all over London to drink the water. It was suggested that it was precisely the people who were supplied with that water were the ones that died. A clergyman came up and drank the water. It was very noticeable he drank brandy with the water, for it was thought brandy corrected the influence of the water. We, of course, know it does not.

This gentleman drank brandy in his water and ate a mutton chop, and spoke of his brethren who had died, and then he went home and died. It was afterward found this water became sparkling because of matter from the sewers from the neighboring hospital. This demonstration was carried on, and it was found the amount of cholera in all these subsequent epidemics in London was in precise relation to the water supplied with animal matter. In London there are two water companies. During the first epidemic the Lambreth Company was the purer. About 137 per 10,000 of the population supplied with that water died with cholera, and only about 67 per 10,000 of those supplied by the other company. The experiment was so large and complete there could be no mistake about it. Houses side by side were supplied by companies furnishing water of various degrees of purity. By the time the second epidemic appeared, the first company went a great deal higher up for the water supply, and the water was much purer. The proportion of deaths then was just inverted. It now had thirty per cent. less deaths than the company formerly more innocuous. A very ghastly experiment.

Now, comes the next fact. In 1866 cholera broke out in East London. I had no skilled, expert hand, but was firmly convinced from study that its cause must be water and nothing but water. I got, then, a very well known gentleman to go down and look at the companies' works, and he came back and said, "There is nothing there. The engineer says the water is the ordinary water supplied in the ordinary way and there has been no change." The same result followed an investigation on the second day, but on the third day I found "pump and repair" on the books. Well, to make a long story short, I found one huge pump had been out of order and all the water was pumped directly into the district, that was affected with cholera, without passing through the filter bed. They were supplied with this water two days before the cholera broke out. Well, that was a very striking fact. Then we found it was only people of that district who were suffering with cholera. London escaped, for the district supplied by each company is limited by act of Parliament, but in five weeks 16,000 people were taken with cholera and 6,000 died. We will now trace how the cholera got into the water. A ship had come by the well known route from Alexandria with *only* six or eight cases of cholera on board. The vessel was put in quarantine, the patients put in the hospital and we had almost forgotten it, but when this occurred we traced these cases, and found one had become almost well and went to a small town. And, there only 16,000 people were killed. But, then came this other fellow, who was discharged as convalescent and had gone to a cottage on the river, the sewers of which connected with the river. He was taken with relapsing cholera. Well, the man infected the river just at the very moment the water was taken from this point, in consequence of the breaking down of the pump, and distributed over the town. Thus, one unhappy man and unhappy engineer combined to kill all these people, and all got off scot-free. I am satisfied we can never have an attack of epidemic cholera in England again. Our water supply is not perfect and we may have limited outbreaks of cholera, but our water supply now places us in much better shape. We have no fear, and can have no fear, of cholera in Great Britain.

The great seats of cholera epidemics have been Spain and Italy. And everybody knows, of course, of Hamburg. In Hamburg they knew they were drinking the filthy and impure Elbe water. But Hamburg is a free municipality, sufficiently under control of the citizens to leave them free to do a good deal of evil and not to do a great deal of good. The greatest mortality rate in Hamburg was down near the river, while further away from the river the mortality rate was much less. Hamburg is as absolute a demonstration of the water-born theory of cholera as the world has ever seen. Also, the recent outbreak, in the dead of winter, near Berlin, where cholera broke out in a lunatic asylum, was a mystery. But, it was found a single person had come on from Berlin. In Hamburg, by the way, they have a most beautiful system of irrigation. Charming! And the engineers have so arranged matters that the sewerage all passes out the same way, into the river. Just below the outlet of the sewerage is the intake of the water supply. And, to make things worse, this being in the dead of winter, everything was frozen and the matter was taken up by the water supply very nearly pure. So, three or four days after the gentleman came to the hospital from Berlin, they had a tremendous outbreak of cholera. That is, also, the history of all Paris outbreaks. In Paris, during the warm season, a notice is put up stating, the water now is mixed and you are only to drink such and such water. But, a great many people cannot afford to drink from that supply, and they always have outbreaks of typhoid and cholera in Paris, caused by the impure water supply, and it is stopped as soon as the water supply is changed. And I may say, if it is supplied with pure water they will never have another attack of cholera in Paris. Marseilles, Toulon, are in the same condition. In Marseilles impure water is taken in, and they pass it out again, running great quantities over the streets to clean them. But cleaning the streets will not stop cholera if you are drinking impure water. After heavy rains, cholera is usually diminished; but after heavy rains in Marseilles it was increased because the unhappy people were drinking the water from rivulets. That was the cause of the cholera in 1866. Another striking case, perhaps more striking, was in Naples. I saw a little paragraph, stating we are all very much afraid of cholera and we are taking every precaution at hand. A few cases have broken out. We are pouring large quantities of carbolic acid in the cess-pools, and, odd to say, and disagreeable enough, our drinking water all tastes of carbolic acid. What happened was, cholera came, and there were 70,000 deaths, the most calamitous and vast catastrophe of modern times. The doctors were attacked and assassinated. They have since taken better precautions than religious processions or attacking the doctors. They have obtained pure water from beautiful aqueducts. Naples is immune from cholera. By a coincidence, little outbreaks occurred, and at the precise period when the aqueduct was being repaired, and the moment it was repaired the cholera ceased. Naples, from being the most susceptible to cholera, is now one of the most immune, as any great city may become when you compel your municipalities to do their duty, to supply pure water, pure air and pure soil. I shall not now detain you furiher than to point out this, that the latest results of microscopical examination and bacteriological research fully confirm this vast mass of evidence, collected from every city, every country, every epidemic of the last half century. Whereas, clinically and statistically, we can say on a basis of facts and figures, there never has been a country experience an outbreak of cholera, where the cause cannot be traced to the distribution of infected water. That is a matter of clinical observation. So, we may say scientifically now, we thoroughly understand why that is. We know the characteristic cause of cholera is a bacterium, which has this simple property: It is not in itself, and while fresh, very poisonous; that it is not an anaërobe but is an aërobe, and its favorite method of development and propagation is in soil and water. The cholera bacillus is not easily communicated in the fresh stage, and it is not easy to infect guinea pigs or other animals with it, but if you cultivate it or let it cultivate itself in water, it becomes very effective, and becomes more virulent as it is multiplied in soil and in water. It is interesting, by the way, to notice it is difficult to infect healthy guinea pigs, but if you dose them with brandy or whisky and make them unhealthy, or if you give them opium, they are easily infected. Acid drinks, sulphuric acid and citric acid, have been shown to be very useful as preventives. So, all these experiments confirm the broad facts, which we have been able to learn from the examinations, and the final result is, if we can exert our influence upon State Boards of Health to bring home a sense of responsibility to the executive officers of State, we shall be able to boast as a profession that we have in our own generation pointed to the extinction of two great plagues of mankind.

